# Legal mobilization and anti-fluoridation campaigning in post-war Britain

**DOI:** 10.1111/jols.70048

**Published:** 2026-02-24

**Authors:** Janet Weston

**Affiliations:** Centre for History in Public Health, London School of Hygiene and Tropical Medicine, London, England

## Abstract

The fluoridation of public water supplies to improve dental health is often cited as an example of an ‘intractable controversy’ in public health, reflecting deeply held principles about rights and the public sphere. This article examines legal mobilization to prevent fluoridation in Britain, from the first pilot studies in the mid-1950s through to *McColl* v. *Strathclyde* in 1983 and the subsequent Water (Fluoridation) Act 1985. I argue that efforts to evade the objections of anti-fluoridationists helped to create an ambiguous legal position from the outset, generating an opportunity for campaigners to use the law to stop fluoridation schemes. I show that, despite there being no decisive legal judgments in their favour, the legal campaigns of anti-fluoridationists were remarkably successful in terms of their indirect effects. Finally, building on recent work that highlights legal mobilization by conservative causes, this historical case study offers insight into the rights claims of a politically diverse populist movement.

## 1 Introduction

The fluoridation of public water supplies is often characterized as an ‘intractable controversy’ in public health, in which people develop strong views based on deeply held principles and are therefore ‘impervious’ to new scientific evidence.^1^ Minds can rarely be changed. The earliest organized efforts to add fluoride to public water supplies to reduce dental caries date from the 1930s in the United States (US), and the protests that fluoridation efforts prompted there in the decades since have been well documented.^2^ Indeed, it remains a hotly contested public health measure in the US, with Utah’s fluoridation ban in early 2025 receiving the praise of the Health & Human Services Secretary Robert F. Kennedy and the condemnation of national health associations.^3^ In Britain, fluoridation has been almost as controversial for almost as long. Despite consistent and decades-long government support for fluoridation, local efforts to introduce it have often met with vociferous objection. As of 2022, only 10 per cent of the population in England (and nobody at all in Scotland or Wales) was consuming artificially fluoridated water.^4^

This article examines the uses of the law to try to prevent fluoridation in Britain, from the earliest pilot schemes in the 1950s through to the 1980s. Successive governments relied on general powers within the National Health Service Act 1946 (NHS Act) and National Health Service (Scotland) Act 1947 (NHS (Scotland) Act) to encourage fluoridation, but this was widely known to be ‘shaky’ legal ground.^5^ There was a serious lack of clarity over whether the initial fluoridation schemes were ‘experiments’ or ‘demonstrations’, which affected their statutory basis, and in either case legal opinion was divided as to whether tap water with or without artificial fluoride was ‘wholesome’ as required by the various Water Acts. Dedicated primary legislation was assumed to be the best solution and draft bills were prepared, but no government was willing to introduce such a measure. Legal action in Andover and Watford took advantage of the resultant uncertainty and helped to halt and discourage fluoridation in the 1950s and 1960s, with another case on the horizon in Yorkshire in 1978. Finally, Strathclyde Regional Council (SRC) faced in court the ire of Catherine McColl, variously described in the press as ‘a toothless Glasgow grandmother’,^6^ ‘an edentulous 69 year old’,^7^ and a ‘committed Communist’ who vehemently opposed their plans for fluoridation.^8^ This long-running case concluded in 1983 with the judgment that the fluoridation proposed by Strathclyde was unlawful, forcing the government to take action. The Water (Fluoridation) Act 1985 aimed – rather too late – to resolve the legal position once and for all.

Building on rich histories of anti-fluoridation campaigns in Britain alongside original archival analysis, this article begins by tracing the origins of the ambiguous legal position that fluoridation came to occupy, and the barriers that prevented decisive action to resolve it. This includes a brief overview of the introduction of fluoridation schemes and the contours of the debate that they prompted. This debate was to some extent a collision between scientific expertise and other more popular or ‘common-sense’ knowledge claims about, for example, fluoride as a poison.^9^ However, as Glen O’Hara argues in his comprehensive history of the fluoridation debates, this was not the whole story.^10^ The conflict was underpinned by competing claims about rights and the public sphere, with anti-fluoridationists unequivocally presenting fluoridation as a moral issue. The most significant factors behind fluoridation’s uncertain legal position were, I argue, an initial failure within Whitehall to appreciate that these moral objections might lead to legal scrutiny, followed by the unwillingness of successive governments to engage with what had rapidly become a controversial issue. In the context of a fractured post-war state, and against the wishes of many Ministers of Health, British governments declined to enter the fray and inadvertently created a clear opportunity for anti-fluoridation activism through legal channels.

I then turn to this activism, which I understand as a form of ‘social movement legal mobilization’. By this, I mean ‘the use of law in an explicit, self-conscious way through the invocation of a formal institutional mechanism’,^11^ by ‘a collectivity acting with some degree of organization and continuity partly outside of institutional channels for the purpose of promoting or resisting change’.^12^ Anti-fluoridationists lodged at least four legal challenges to fluoridation in Britain between the 1950s and the 1980s, and I argue that these were remarkably successful in spite of what looks rather like failure. Though one aspect of this failure is an unusually clear example of government ‘striking back’ through the Water (Fluoridation) Act 1985 to eliminate the effects of an unwelcome judicial decision, the example of British anti-fluoridationism also demonstrates the potential for apparently unsuccessful legal mobilization to deliver powerful ‘indirect’ effects.^13^ Analysis of this social movement legal mobilization also offers context and insights in relation to contemporary concerns, including the rapid spread of anxieties about new interventions, the place of parliament when it comes to difficult moral questions, and trust in the machinery and personnel of government. Legal mobilization offered one route to raise these fears and to try to re-establish trust, where an official position on the moral questions was lacking.

Finally and more briefly, I consider the nature of the anti-fluoridation movement. Anti-fluoridation campaigns provide an example of social movement legal mobilization that draws on the language of rights claims, but cannot be characterized as straightforwardly ‘progressive/liberal’ or ‘conservative’. Instead, they can be seen as part of the ‘grey zone’ described by Paul Blokker, where such distinctions are a poor fit.^14^ Building on recent critiques of the assumption that social movement legal mobilization is always oriented towards progressive goals associated with social justice, this adds to Carol Harlow and Richard Rawlings’ history of ‘pressure through law’ that includes much more politically diverse causes.^15^ Anti-fluoridation campaigning was part of the post-war explosion in both Britain and the US in single-issue or ‘cause’ groups displacing traditional party politics,^16^ and it united people from across the political spectrum. Anti-fluoridationists were brought together by their deeply felt hostility towards forms of authority that were failing to protect them, intruding increasingly into their lives while ignoring their rights, needs, and wishes. Those involved in anti-fluoridation legal mobilization, including dentists, local councillors, trade union organizers, housewives, peers of the realm, and some scientists, used the tools of scientific argumentation, alongside moral claims about the state and the rights of its citizens, to critique the trajectory of the post-war nation. In this light, anti-fluoridation legal mobilization can be seen as a precursor of more recent populist action.

## 2 The Emergence of Legal Ambiguity

Legal mobilization theorists have argued that legal opportunity, whether structural or contingent, is essential if social movements are to take up this form of action.^17^ For anti-fluoridationists, legal opportunity structures were in their favour, not only in the form of relator actions and, later, legal aid, but also through the uncertain legal position that fluoridation came to occupy. To understand how this came about requires a return to the first days of fluoridation in Britain.

As O’Hara notes, dental health and particularly the state of children’s teeth became a pressing problem for the newly nationalized health service, particularly as sugar rationing came to an end in the early 1950s.^18^ News of fluoridation schemes in the US prompted the deployment of a small research party from Whitehall across the Atlantic in 1952. Their enthusiastic report recommended that fluoridation should be adopted in Britain, subject to some local studies to confirm that the same positive results would be obtained despite any differences between US and British populations in terms of diet, dental hygiene, water consumption, and so on.^19^

The Ministry of Health (MoH) set up a committee to oversee the implementation of these studies, and began looking for towns with naturally low levels of fluoride, an isolated water supply, and a large enough population to generate meaningful information about fluoride’s effects. The enthusiasm of the local authorities was also an essential practical requirement, and there had been early signs that this might be forthcoming since both Kilmarnock in Scotland and Anglesey in Wales had eagerly requested permission to try out fluoridation even before the idea of study sites was fully formed. Perhaps influenced by this zeal, Chief Medical Officer Sir George Godber had initially assumed that the MoH would have a wide choice of study sites, since ‘our main difficulty will be in holding people back’.^20^ However, the emergence of hostile public opinion soon made it clear that this would not be the case, and early ambitions to select towns in both northern and southern England, and with hard and soft water, fell away. Public opposition in Norwich prompted the local authority to withdraw their initial agreement to participate in 1954.^21^ Darlington then also withdrew in January 1956, following a protracted campaign and amid much local ill-feeling.^22^

Public opposition to fluoridation drew on a wide array of concerns and priorities. These included an emphasis on the sanctity of the body and individual choice over its care, fears about unreliable modern science and the potential for fluoride to cause as-yet-unknown illnesses, objections to the idea of enforced ‘mass medication’, objections to the dissemination of possible environmental toxins, impulses towards self-sufficiency and a small state, hostility towards big industries (whether these were harming dental health with unhealthy foods or trying to find ways of disposing of dangerous chemical waste products), and suspicion that important individual freedoms or responsibilities were under threat.^23^ This array of complaints can be grouped under two broad headings: that fluoridation was in some way dangerous or harmful, and that it was morally wrong.

Both strands of objection spread rapidly from the 1950s onwards, demonstrating that the contemporary problem of health-related misinformation is not exclusive to the age of social media. As Charlotte Sleigh documents, anti-fluoridation arguments were packaged and disseminated by a small number of key figures for whom the flames of ‘genuine concern’ were often fanned by a sense of lost (or always low) status.^24^ Members of the British Housewives’ League were at the vanguard of anti-fluoridation messaging, which resonated with existing concerns about scientific ‘progress’ and the reliability and direction of central and local government. Local campaigners including League members sowed doubts and fears about fluoridation quickly and effectively, with trusted local figures and powerful personal stories winning over hearts and minds in towns where fluoridation was proposed. Such strategies were particularly important given that both sides of the debate drew on expert opinion, citing scientific publications and apparently eminent expertise in support of their case. Without an in-depth understanding of numerous relevant research fields, most people – including ‘experts’ such as doctors, dentists, Medical Officers of Health, and other officials – had to use ‘social discrimination’ to decide who and what to believe, with divergent results.^25^

The MoH knew that the British Housewives’ League and others were likely to object to fluoridation, but showed little concern.^26^ Slightly more troubling to the MoH was the fact that, as soon as test sites for fluoridation in Britain were first envisaged, there were some doubts about their legal basis. Dame Enid Russell-Smith, a senior civil servant at the MoH, noted as early as May 1953 that ‘the legal position is, to say the least of it, shaky’.^27^ Section 29 of the NHS (Scotland) Act 1947 empowered local health authorities to ‘make such provision for conducting, or assisting the conduct of, research as appears to the authority desirable for purposes relating to their functions’, which seemed sufficient for the study site in Scotland.^28^ For England and Wales, Section 16 of the NHS Act 1946 would suffice; it conveyed a similar power directly to the Minister. Questions remained surrounding the powers of water providers to add extra substances to water that was already ‘wholesome’ and whether ratepayers’ money should be spent on such projects.^29^ However, civil servants and ministers were willing to set these worries aside, resolving them for the time being with an indemnity for water providers in the study sites in case of legal action ‘brought against them for “polluting” the water’.^30^ It was assumed that the position would be clarified by new legislation before the five years of pilot studies came to an end. The studies launched in 1955–1956 in Kilmarnock and Anglesey, and in Watford and Andover in the south of England, selected on the very pragmatic basis that they were all willing and able to participate.

Mounting opposition within some of these study sites prompted closer examination of the legal position. Watford’s Town Clerk sought urgent advice in 1956, having received letters from ‘a resident whom we believe to be a solicitor and to be well advised’.^31^ Russell-Smith observed that within the MoH, ‘lawyers do seem to be expressing doubts now which were not at all forcibly expressed when they were consulted back in 1952’.^32^ Appreciating that an awkward situation was rapidly taking shape and that primary legislation might be needed sooner rather than later, the Minister of Health requested the informal views of the Attorney General (AG) and received unwelcome news. Water providers, according to the AG, clearly had ‘no power to add fluoride’ to already ‘wholesome’ water at the study sites.^33^ Perhaps more immediately challenging was his view that, since the study sites were now being described as ‘demonstrations’ and not ‘experiments’, they did not fall under the general powers for health research. Local authorities could perhaps request to fluoridate their water under general powers to take action to prevent illness, as long as water providers were indeed legally able to fulfil that request. However, as the AG concluded, if the studies had been undertaken on this basis, then they should not have been funded by the Treasury but by the local authority itself – which then raised the question of whether local authorities were empowered to pay for such activities.

The MoH thus found itself in a rapidly deepening quagmire, facing the prospect that its studies were unlawful on several grounds. The AG was correct to note that the words ‘research’ and ‘experiment’ were falling out of favour.^34^ This was largely the result of anti-fluoridation arguments about the wrongs of medical research or experimentation carried out on involuntary subjects. As the well-advised Watford correspondent H. J. L. Monk pointed out, the ‘Nuremberg Standards [sic]’ were unequivocal ‘that voluntary consent is absolutely essential’.^35^ To ‘experiment’ on a group of people who had not given their consent and had no way to opt out of the research contravened fundamental and newly specified principles of medical ethics and was redolent of Nazi atrocities. The framing of fluoridation as experimentation on unwilling subjects had appeared in a 1954 House of Lords debate,^36^ and in a ‘Voters’ Veto’ petition from Darlington in 1955:

We do NOT CONSENT to the putting of FLUORIDES in the water supplied to our homes, and REQUIRE YOU, AS OUR REPRESENTATIVE(S) to oppose in the Council until it is dropped this proposal to use us as the unwilling subjects of a medical experiment, in contravention of our rights as citizens and as human beings.^37^

As anti-fluoridationists noticed with some suspicion, amid this controversy, the word ‘demonstration’ gradually replaced ‘research’ or ‘experiment’ in official pronouncements.^38^ This linguistic shift did little to instil confidence in those who were already dubious about the reliability and accountability of government, and its capacity (or desire) to protect its citizens’ rights.

From the outset, civil servants had recorded that the study sites were not really ‘experiments’ in the sense of ‘checking of the validity of the American findings’, but rather ‘shop windows or demonstration centres’ to show how fluoridation could be done and with what benefits in Britain.^39^ The choice of study sites was more practical than scientific, but this reflected the messy reality of public health research and practice more than any clear-cut sense that the sites would provide logistical insights and reassurance rather than the ‘results’ of experimentation. It was unfortunate that the word ‘experiment’ had continued to circulate, and that the (presumed) legal basis for the four ‘demonstrations’ was the general powers within the NHS Acts to carry out research. When asked directly in 1959, an MoH official clarified that ‘the distinction between a demonstration and an experiment lies in the degree of confidence with which the outcome can be predicted. If with certainty, it is a demonstration; if without, it is an experiment.’^40^ As far as the MoH was concerned, ‘the experimental stage has long since passed’ for fluoridation, and the four study sites were thus ‘demonstrations’. The test sites were therefore *not* deemed to be unethical medical research. A terminological adaptation to avoid moral censure thus exacerbated the legal problem. If the studies were not ‘experiments’, then they were not being carried out under general provisions in the NHS Acts for research; however, if they were not research, then they should not have been funded by the Treasury. In any case, as the AG’s informal advice indicated, water authorities simply had no power to add fluoride to their supplies, whether for research *or* the prevention of illness.

Civil servants across health and local government believed that the solution was urgent primary legislation. Such a plan was considered by the Conservative Cabinet in 1958, and steps were taken to prepare a draft bill, but O’Hara notes a distinct lack of appetite among members of parliament (MPs) to proceed, given that it would have been fiercely opposed by anti-fluoridationists within parliament and beyond.^41^ The government’s stability and popularity had been shaken by Prime Minister Anthony Eden’s resignation the year before, and a controversial bill would have presented a threat to their efforts to rebuild. This lack of appetite persisted. After the general election in 1959 and the return of a decisive Conservative majority, the idea of a private member’s bill was briefly entertained before the Home Affairs Committee agreed to introduce a fluoridation bill as soon as time permitted; it never did.^42^ When the problem resurfaced again in 1962, as the ‘demonstrations’ drew to a close, the timing was again far from perfect. The drug thalidomide had just been withdrawn amid mounting evidence that it caused severe birth defects, raising new concerns about the reliability of innovative medicinal products and the science that vouched for their safety. Once again, government popularity was in decline, and the Conservative Party was internally fractured and fractious. Though the Law Officers for both England and Wales and Scotland provided extremely pessimistic advice as to whether fluoridation could be implemented lawfully under existing statute, primary legislation was once again avoided.^43^

The official government position on fluoridation therefore came to tread a rather fine line. Positive results from the ‘demonstration’ sites were published in 1962, accompanied by the announcement that ministers were

ready to approve, under Section 28 of the National Health Service Act and the corresponding Section of the Scottish Act, proposals from local health authorities to make arrangements with water undertakers for the addition of fluoride to water supplies which are deficient in it naturally.^44^

This relied on general powers to take action to prevent disease, and did not address the question of whether water providers could lawfully add substances to ‘wholesome’ water. Parliamentarians in favour of fluoridation simply insisted that the legality of any future fluoridation scheme was a matter for the courts.^45^ It was accepted in Whitehall that the door was very much open to legal challenge, and local authorities seeking approval for fluoridation schemes were once again provided with an indemnity.^46^

One key explanation for the absence of more decisive governmental action, as O’Hara argues, was the fragmented state in post-war Britain; post-war politics was ‘extremely turbulent, unstable, and even at times apparently incoherent’.^47^ Promoting fluoridation legislation would require a determined and confident government, and this was lacking. Though numerous incoming Ministers of Health were initially in favour of legislation, they were invariably persuaded by their colleagues and advisors not to take on such a controversial battle. Enoch Powell as Minister of Health – a firm advocate for fluoridation who publicly branded its opponents ‘cranks’^48^ – concluded that a government-sponsored fluoridation bill would ‘only be accepted as such by my colleagues and got through Parliament against a background of public agitation *for* it’.^49^ This was not only about the value of following popular opinion; it also reflected conflicting views *within* parliament about the proper role of the state when it came to health, and whose rights – individuals who disliked fluoride, communities whose representatives voted in favour of it, ratepayers, parents, children whose future caries could be prevented – should be prioritized. Despite some efforts to drum up public support, this unresolved conflict along with the uncertain legal status of fluoridation left pro-fluoridation advocacy rather muted.

As this begins to suggest, the difficulty of a fragmented state dealing with a controversial issue was exacerbated by the very particular tangle that had been woven around fluoridation. The most difficult anti-fluoridation arguments to dislodge, as was noted then and since, were moral ones.^50^ Many campaigners consistently emphasized that this was a ‘*moral question* … No man can force another person to have what they don’t want [or] compel others to consume a substance against their will.’^51^ Science could not readily answer the moral questions that fluoridation raised, for all that scientific research was constantly summoned by both sides. This remains an underappreciated aspect of public health policy, where claims to be simply following scientific research obscure the many decisions that must be made before ‘science’ becomes action. As social scientists have argued in relation to politicians’ claims to be ‘following the science’ during the worst of the COVID-19 pandemic, such claims are a way to avoid blame and can be deeply damaging to democratic institutions.^52^ Noting that ‘the strongest objections to fluoridation are the moral ones’, one civil service commentator observed in 1967 that ‘legislation showing that the procedure had the support of Parliament would be the most effective answer to this’.^53^ There was, in other words, an opportunity to acknowledge that significant policy decisions were the responsibility of parliament and to address openly the moral as well as the scientific questions. However, it became increasingly difficult to change position as fluoridation continued to take place; legislation risked acknowledging that it may have been introduced unlawfully, as well as stirring up difficult points of principle.

Reluctance to address the controversy became a vicious cycle. The uncertain legal status of fluoridation had been created and sustained by a failure to appreciate at the outset that the practice was going to raise questions about consent, choice, and the proper exercise of state power, and by persistent efforts to evade rather than engage with public opinion. Yet, by declining to take a firm position, successive governments generated an increasingly difficult situation for any local authority interested in fluoridation, and for anyone who sought to advocate publicly for its wider adoption. How could any official give fluoridation their full-hearted support, when opponents could immediately point out that it might not be lawful? As time went by, however, the Law Officers cautiously opined that ‘a Court would be unlikely to rule, after so many years, that the *vires* of the Water Acts were unsound’.^54^ In Scotland in 1983, this was proven wrong.

## 3 Legal Mobilization in Action

*McColl* v. *Strathclyde* is notorious in a small way for two reasons: its duration and its cost to the public purse. Both were record breaking for Scotland; the case involved mountains of technical evidence requiring over 200 days in court, and both parties were funded by the state, through legal aid and the indemnity provided to all local authorities implementing fluoridation, with total costs exceeding £740,000.^55^ The case has been largely disregarded in terms of its legal significance, with one or two exceptions,^56^ since the only aspect in which it found in favour of the petitioner was quickly superseded by the Water (Fluoridation) Act 1985. Seen as part of the anti-fluoridation movement’s legal mobilization, however, this case – like its precursors in England, which did not make it to court – offers valuable insight into how social movements in post-war Britain used the law, and with what effect. I argue that, despite no durable legal victories to speak of, this legal mobilization was in fact remarkably successful. It exemplifies the wider impacts of legal mobilization, as presented by Michael McCann and others, where ‘indirect’ effects are just as important as the actual outcomes of litigation.^57^

Campaigners against fluoridation included national figures such as author Barbara Cartland, as well as teachers, clergy, housewives, business owners, retirees, dentists, and some parliamentarians, including Labour peer Lord Douglas of Barloch and Conservative MP Ivan Lawrence. Though anti-fluoridation campaigning was for most a single-issue protest, for some it was part of broader efforts to address socio-political concerns. As historian Amy Whipple observes and as the MoH noticed at the time, the British Housewives’ League was an essential driving force behind anti-fluoridation activism in Britain,^58^ and its annual report in 1956 described one local battle against fluoridation as ‘a minor conflict in the great war of our time, which is concerned with freedom of choice and of action’.^59^ Associated with this was the League’s members’ deep anxiety that bureaucratic power was increasing exponentially through the broad and vague powers being given to Whitehall, and that the nation, as they saw it, was sleepwalking into despotism.^60^ For the petitioner Catherine McColl and others in Glasgow, anti-fluoridation campaigning seems to have been one part of an array of activism oriented towards the protection of socio-economic rights.^61^ Along with serious doubts about the promises made by modern science – particularly when that science could not provide absolute certainty and agreement – anti-fluoridationists evidently also had concerns about the state institutions seeking to make decisions about fluoride. These concerns can be seen as part of a decline in public trust that began in the 1950s and, in some analyses, has in the twenty-first century reached crisis point.^62^ To test and perhaps re-establish the trustworthiness of their governing institutions, anti-fluoridationists sought to use one of the systems through which nations try to engender trust: the law.

The idea of legal mobilization against fluoridation originated with the British Housewives’ League. The League questioned the legal basis of the proposed study sites and was ‘further inflamed’ by the news that the participating local authorities were to be indemnified by the MoH, which was seen as confirmation that to fluoridate was ‘to flout the law’, and that all involved knew this perfectly well.^63^ In Watford, as mentioned above, the Town Clerk began receiving letters opposing fluoridation plans from someone who appeared to be a well-informed solicitor, in which more specific legal questions were asked.^64^

The early involvement here of a (suspected) solicitor endorses the view that the involvement of legal professionals with a social movement renders legal mobilization much more likely to occur.^65^ That said, at the site of the first legal challenge in Andover, the movers and shakers were not lawyers. One key figure was in fact a dentist, T. McClelland, and another was local shop owner and former mayor Olive Harvey.^66^ As respectable local figures who could disseminate anti-fluoridation messaging, and whose views on fluoridation would carry weight, better representatives could hardly be chosen. Soon after fluoridation in Andover began in the summer of 1956, McClelland, Harvey, and others began fundraising for legal action, and in July 1957 a relator action was initiated.^67^ This sought a declaration that fluoridation in Andover was *ultra vires*, an injunction preventing the council from adding fluoride to the local water supply, and a declaration that the council could not spend ratepayers’ money on fluoridation equipment.^68^

The necessary certificate for the relator action to proceed was received from the AG in January 1958, but local elections in Andover in May disrupted progress. Leading anti-fluoridationist Harvey defeated the mayor in the election, and the chair of the Public Health Committee – another staunch supporter of fluoridation – was also ousted, transforming the balance of views within the council. This meant that, at the monthly council meeting on 1 July 1958, it was agreed to end fluoridation with immediate effect. Campaigners were keen to ensure that it could not be reintroduced if local power shifted again in future, and the case was finally stayed on the basis that the local authority would have to give six months’ notice if they ever wanted to reintroduce fluoridation, to allow proceedings to recommence.^69^ Though the anti-fluoridationists did not have their day in court, it was a victory nonetheless; fluoridation ceased, and the idea that this was a direct result of the legal action persisted within MoH files and among campaigners, all of whom recognized that the lack of a court judgment only sustained uncertainty as to whether fluoridation was lawful.^70^

As soon as Andover withdrew its fluoridation scheme, MoH papers noted that opposition in Watford became ‘more active’ as campaigning energies transferred there.^71^ Anti-fluoridation campaigning also gained important structure and resource with the formation of the National Pure Water Association (NPWA) in 1960, with Lord Douglas of Barloch as its president and five of his parliamentary colleagues as vice-presidents. The NPWA quickly ‘obtained a copy of the Counsel’s Opinion in the Andover case’,^72^ and its concerns about the legal position of fluoridation were further boosted when Lord Douglas of Barloch reportedly received confirmation from the Minister of Health in post during the ‘demonstrations’ in the 1950s that fluoridation was unlawful.^73^ As it became clear that fluoridation was going to continue in Watford beyond the ‘demonstration’ phase, a writ was served on the local authority in the name of Harry Foxwell, a local primary school teacher who was also a member of the NPWA, seeking the same injunction and declarations as in Andover.^74^ After two years of slow progress, this was withdrawn in July 1965. As far as the MoH was concerned, this was thanks to several judgments in other countries in favour of fluoridation that seemed to undermine the relator’s claims: one in Ireland, confirmed on appeal, and one in Aotearoa New Zealand.^75^ The public statements of the NPWA gave a different story, insisting that the case had been discontinued ‘solely on account of expense’ and emphatically not because the central issues had been decided elsewhere, thus retaining the option to resume the challenge in future.^76^ Fluoridation seems to have continued in Watford until 1989, when ‘an over-dosing accident’ saw excessive amounts of fluoride added to the water supply and a hasty halt called.^77^ It does not seem to have resumed since.^78^

Even though the action was withdrawn and fluoridation continued in Watford for decades, the case chilled official efforts to encourage fluoridation elsewhere. Campaigners continued to call into question the lawfulness of fluoridation throughout the 1960s and 1970s.^79^ This included warning water authorities and local councils of their possible liabilities, to good effect.^80^ Many local officials struggled to respond to such claims and were left ‘feeling extremely angry and frustrated’, as the Keighley medical officer wrote of his efforts to implement fluoridation.^81^ The British Waterworks Association circularized its members on several occasions in the 1960s, stating that they could not advise members to participate in fluoridation unless and until they received ‘further clarification of the position of statutory water undertakers by Government action accompanied by any necessary legislation’.^82^ Though they revised their position after a meeting with the Minister of Health and declared themselves ‘neither for nor against’, this was hardly a ringing vote of confidence.^83^ Birmingham and some parts of the north east were the only locations to introduce fluoridation on any significant scale.

A national refocusing on preventive health in the mid-1970s saw renewed attention paid to fluoridation, and the NPWA obtained their own counsel’s opinion, which confirmed, once again, that the addition of fluoride certainly seemed to go beyond the powers of water authorities.^84^ ‘The majority of Water Authorities have decided that they will not consider requests for new fluoridation schemes’, the NPWA could eagerly report in 1979, ‘until the legal doubts have been resolved.’^85^ The next in line for a legal battle looked likely to be Calderdale Borough Council, with a writ served on Yorkshire Water Authority in August 1978,^86^ but it was in relation to Strathclyde that a court was finally called on to consider the question. Trouble in the west of Scotland had been brewing for some time, ever since Kilmarnock had withdrawn fluoridation as soon as its role as a ‘demonstration’ site ended in 1962.^87^ Amid local protests, practical impediments slowed the progress of fluoridation across Scotland’s many small water plants and rural communities.^88^ SRC was able to overcome this in 1977 when the Lanarkshire Health Board, the last of the four health boards covering areas for which SRC was responsible for water supplies, finally voted in favour of fluoridation. SRC promptly sought approval (and indemnity) from the Scottish Home and Health Department to begin work on adding fluoride to all of its water supplies, which would eventually reach about half of Scotland’s population.^89^

This momentous decision prompted one of Strathclyde’s Labour councillors, James Wray, to warn that an interdict would soon be sought from the courts to prevent SRC’s plans.^90^ Along-side Wray, who continued to appear at press conferences with the petitioner Catherine McColl, a network of supporters from the wider anti-fluoridation movement emerged including the Scottish Housewives’ Association, the Scottish National Pure Water Association, and the Strathclyde Campaign against Fluoridation, with Rosemary Young at its helm and funding its coffers.^91^ Young also acted as secretary for an organization called Concern International, whose patrons and advisors included leading international voices against fluoridation, many of whom gave expert evidence in the trial. Concern International was interested in ‘environmental and population protection’,^92^ and, for those opposed to fluoridation, both environment and people needed protection from powerful forces that cared little about them. In Young’s summary of the trial for Concern International supporters, she described McColl as a ‘thinking human being who cares for her people’ and recounted her opening testimony ‘that people were more precious than the Koh-I-Noor Diamond’.^93^ For Young, the ‘courage of this wee lady in saying NO to the Giants who put profits from chemical waste before the health and welfare of innocence people … has already made history’. A lack of trust in governments, institutions, and science, particularly among marginalized populations, has been associated more recently with lower uptake of health measures such as vaccination, and the distrust felt by anti-fluoridationists several decades earlier is clear.^94^ The insistence that citizens required protection that government had failed to deliver – leaving it to a wee ‘Glasgow grandmother’ instead – was met only occasionally and less publicly by counter-claims that the state had a duty to protect the wellbeing of the community as a whole, or to save future generations from dental pain.^95^

McColl relied on claims that fluoridation was dangerous to health and that it was a medicinal product being dispensed without a proper licence, as well as the now-frequent claim that it was *ultra vires*. In his judgment, Lord Jauncey found that there was no evidence of harm to health from fluoridation; his conclusions regarding its medicinal status were less clear cut, as David Shaw notes, but ultimately found no breach of the Medicine Act 1968.^96^ *McColl* v. *Strathclyde* represented a victory for anti-fluoridationists only thanks to the finding that fluoridation was indeed beyond the powers of the local council. Here, Lord Jauncey reached the conclusion that had been long anticipated within government: that water without fluoride added was already ‘wholesome’, as required by the relevant Water Acts, and that there was no statutory power for water providers ‘to treat the water which they supplied for such general health improvement purposes as they might from time to time think appropriate’.^97^ Reflecting arguments from anti-fluoridationists that prioritized individual rights and choices concerning the body and matters of health, the judgment noted that ‘if two possible constructions of a statute are available, that construction should be preferred which encroaches to the minimum on the personal rights of the individual’. In other words, ‘the individual’s right to choose how to care for his own body should only be encroached upon by statutory provisions in clear and unambiguous language’. Such language did not exist.

In light of this, the indemnity offered to all fluoridating local councils in Scotland was withdrawn, and fluoridation – such as it was – abruptly ceased.^98^ SRC initiated an appeal and the pressure for government action began to mount, to avoid both the expense of an appeal and the possibility of fluoridation across England and Wales coming to an end too. Of the various options, including legislating to compel fluoridation nationwide, introducing ‘[c]larifying and permissive legislation’ was selected as the best of the bunch: ‘[It] would be more attractive than any other kind to our Back Bench supporters (some of whom have already signed Early Day Motions opposing fluoridation) and no change of Government policy would be involved.’^99^ Despite government determination to act, therefore, legislation remained controversial, and anything more forceful than simply giving water providers permission to fluoridate was a step too far. A bill was announced in December 1983 and achieved royal assent the following November, demonstrating the speed with which a confident new government with a large majority could resolve the long-running legal ambiguities around fluoridation. The fact that it was permissive only, however, left the underlying competing claims about rights, protections, and the role of the state unaddressed.

This was an example of the government ‘striking back’, taking advantage of the sovereignty of parliament and the absence of a codified British constitution to eradicate the effects of an unwel-come judicial decision.^100^ Unsurprisingly, this appeared to some to be profoundly unfair and must have done little to increase trust in public institutions. ‘When you lose, you lose, but when you win, they change the rules’, complained one correspondent to their local newspaper.^101^ Yet since the late 1950s and the relator action in Andover, this was exactly what advocates of fluoridation in Whitehall had been planning. It had long been accepted within the Departments of Health and Local Government that any court finding that fluoridation was unlawful would serve as the necessary impetus for legislation. In the wake of the Water (Fluoridation) Act 1985 – and in light of the decisive conclusions expressed by Lord Jauncey regarding the safety of fluoridation – it seemed as though *McColl* v. *Strathclyde* was an unusually clear-cut example of the limitations of legal mobilization for social movements in Britain.

That said, this case and its precursors south of the border are perhaps more persuasively seen as an example of the ‘radiating’ or ‘indirect’ effects of legal mobilization, in which it is not the judgment itself but its gradual impact on wider society that matters. Despite the Water (Fluoridation) Act 1985, Strathclyde did not continue to pursue its fluoridation plans, and there is still no artificial fluoridation in Scotland today. This step back from fluoridation – surely good news for anti-fluoridationists – was the culmination of decades of legal uncertainty, which left only a very small number of local authorities sufficiently confident to proceed. As well as highlighting genuine legal ambiguities, legal mobilization clearly served as leverage for anti-fluoridationists. As McCann writes, ‘litigation is most successful when it works as an unfulfilled threat, but activists must be willing to follow through occasionally’,^102^ as they did in Andover, Watford, and Strathclyde. Even though the financial burden of defending claims was covered by indemnities, local authorities could see from events in Andover and Watford that a legal challenge from their voters and ratepayers meant a commitment of time and resources, with reputational (and electoral) consequences. Given the very small number of regions that did set up fluoridation schemes throughout the 1960s and 1970s, it seems likely that many water providers and local authorities alike were deterred.

A recent report found that though some fluoridation introduced in the 1960s and 1970s still continues in England, ‘no such schemes have in fact ever been made under the statutory regime initially established by the Water (Fluoridation) Act 1985’.^103^ The reasons for this are complex, and include the financial strain under which local authorities have almost always operated and the privatization of water providers in 1989, following which water companies could argue that it was not in their shareholders’ interests to undertake fluoridation. Anglesey, the last of the 1950s ‘demonstration’ sites, saw its fluoridation quietly withdrawn in 1992 with no public announcement; Welsh Water took the decision at its annual general meeting and declared that it would only recommence fluoridation if required by law.^104^ Notwithstanding these wider structural challenges, the radiating effects of legal mobilization since the 1950s also contributed to creating a hostile environment for fluoridation, with Welsh Water citing in support of the withdrawal the potential legal liabilities. When it came to stopping and preventing fluoridation, legal mobilization was one highly effective strand of the anti-fluoridationists’ approach.

## 4 Rights Claims and Populism

As David Borrett points out in his local history of fluoridation in Andover, and as O’Hara and Whipple also find in their national studies, these campaigns in Britain crossed traditional political lines.^105^ Anti-fluoridation sentiment drew on calls to protect individual freedom of choice in the face of state intervention, typical of conservative or more right-wing philosophies of a small state or libertarianism, but also the organicist or environmental movements that would come to be associated with left-wing politics. Anti-fluoridationists set aside party-political allegiances, with Labour peer Lord Douglas of Barloch, a passionate advocate of natural foods and the protection of the environment, sharing an anti-fluoridation stance with Conservative MP and Monday Club member Ivan Lawrence, who considered it to be ‘the worst act of collectivism one can consider’.^106^ The British Housewives’ League and Scottish Housewives’ Association, meanwhile, were not officially affiliated with any political party, though the League did tend towards the political right (and occasionally far right).^107^ For many women who opposed fluoridation, objections to state encroachment into the domestic sphere through the very water that emerged from the kitchen tap could sit comfortably alongside desires for more natural, organic, or ‘wholesome’ diets that would exclude chemically ‘tainted’ water (and sugary foodstuffs that harmed teeth too).^108^

The involvement of the trade union movement, with its connection to left-wing politics, was notable in Strathclyde in particular. Local branches of the Union of General and Municipal Workers and the Draughtsmen’s and Allied Technicians’ Association advocated against fluoridation in the early 1960s.^109^ By the late 1970s, officials feared that the Glasgow Trades Council was marshalling opposition among unions, and it was reportedly common knowledge that the National and Local Government Officers’ Association was ‘generally opposed’ to fluoridation.^110^ The precise basis for this trade union opposition is unclear, but paying close attention to some of the individuals involved in Scottish anti-fluoridationism begins to suggest an additional perspective, beyond anti-statism or organicism. The petitioner in *McColl* v. *Strathclyde* shared with her local councillor Wray a history of community activism; McColl had been an ‘active trade union official’ according to her obituary,^111^ and Wray a ‘die-hard socialist’ who had organized a rent strike a decade earlier.^112^ For both, anti-fluoridation campaigning may well have aligned with their role in Glasgow’s history of social activism: ‘a long and continuous tradition of struggles over housing and amenities’ dating back to the early twentieth century.^113^ McColl described fluoridated water as a ‘witches’ brew’, and much of her case focused on the harms that it was alleged to cause to consumers.^114^ Clean, safe water was, after all, a fundamental amenity for any community.

This strand of anti-fluoridationism drew on long-standing suspicion in Glasgow that persistent community pressure was required to ensure that public authorities fulfilled their obligations, as well as fears about the safety of fluoridation.^115^ Hostility towards officialdom also emerged strongly in the letters of solicitor Peter McCann, instructed by McColl. Despite – or perhaps because of – having been a civic leader himself in the 1970s, McCann had little trust in the institutions and workings of local government, and though he claimed no animus towards fluoridation specifically, he seems to have been frustrated by the ‘arrogance’ and dismissive attitudes that he encountered among civil servants and MPs.^116^ McCann had long campaigned for the rights of disabled people and went on to work against the closure of a local swimming pool and the transfer of council housing stock to private housing associations,^117^ all of which suggests a desire to promote the interests of the marginalized and to protect public amenities for the good of the people. For this group, legal mobilization against fluoridation was part of a mission to protect social rights and to force local or central government to follow its own rules. It reflected deep distrust of the motives and competence of authorities, and perhaps also a sense of exclusion from the levers of power that could only be rectified through legal challenges.^118^

The fact that the law is used to put pressure on the state in the service of different political agendas should not be particularly surprising. As Harlow and Rawlings demonstrated in the 1990s, pressure groups have historically used legal tactics to serve all sorts of causes, including campaigns against slavery, compulsory vaccination, and animal cruelty; ‘moralizing’ campaigns against drunkenness, prostitution, and other forms of ‘vice’; and campaigns against the privatization of public lands and monuments, to name just a few.^119^ Though post-war social movement legal mobilization has typically been associated with progressive causes, such as those addressing race- or gender-based discrimination, the example of anti-fluoridation tells a more complex story.^120^ Recent scholarship has noted the rise of conservative social movements borrowing the tactics of legal mobilization from progressive groups, and a renewed interest in the US in conservative engagement with the law.^121^ There can be a tendency, however, to present this as a new development and to see only movements working towards progressive social goals as undertaking ‘legitimate forms of legal mobilization’, distinguishing between legal mobilizations that make use of human rights for ‘the advancement of social justice’ and those that strive to ‘sustain hegemonic power’.^122^ It is not clear that anti-fluoridation mobilization fits into either category.

Anti-fluoridationists themselves certainly believed that they were fighting to protect their rights. As Blokker points out, rights claims are not necessarily progressive, and the distinction between ‘liberal’ and ‘conservative’ legal mobilizations is not always evident.^123^ Though legal actions to prevent fluoridation made few explicit rights claims – much to the frustration of some campaigners left baffled by legal procedure and tactics^124^ – the language of rights swirled around them, and was echoed in Lord Jauncey’s conclusions regarding ‘the individual’s right to choose how to care for his own body’. Much more muted were the competing rights claims put forward by those in favour of fluoridation: the rights of ‘the present generation of children’ to benefit from improved dental health through fluoridation, and the rights of the majority who desired (or did not care about) fluoridation to receive it.^125^ These points only emerged years after anti-fluoridation arguments had made the headlines, and lacked the same force. In this debate, initial calls to respect individual rights to choose and collective rights to ‘untainted’ water drowned out belated counter-claims to consider population health and inequities.

Much of this resonates with contemporary debates about health and the role of the state, perhaps most visibly in the US but elsewhere too. Anti-fluoridationists were deeply suspicious of governments, civil servants, and the majority of researchers who found no risks associated with fluoridation. To some extent, this represented a collision between the knowledge claims made by experts relying on scientific evidence and those made by sceptics drawing on more popular or ‘common-sense’ forms of knowledge. However, given the reliance of anti-fluoridationists on scientific research (as long as it supported their concerns), and the inclusion in their number of ‘experts’ such as dentists, researchers, and governors at the national and local levels, this was not the whole story. Objections to fluoridation reflected conflicting visions of whose rights required protection, from whom, and – by extension – the role of the state in dealing with matters of health. Anti-fluoridationists used the language of ordinary people battling to protect their rights from an untrustworthy, arrogant, or incompetent elite – a rhetorical technique that has been associated with contemporary populism.^126^ Though many scholars trace the origins of contemporary populism back to the austerity measures that followed the financial crisis of 2008, against a ‘back-drop of neoliberalism’,^127^ anti-fluoridation campaigns are a significant precursor to these more recent upsurges. An early form of populist legal mobilization, they won support through rhetorical rights claims, even while most of their legal claims were rooted in ambiguities about the statutory obligations of water providers that successive governments chose to ignore.

## 5 Conclusion

As well as providing the first account of legal battles over fluoridation in Britain, this article has contributed to research on social movement legal mobilization. It has shown that the introduction of fluoridation in Britain in the 1950s left considerable scope for legal challenge, thanks to a failure at the outset to deal with the ‘shaky’ legal ground on which fluoridation was introduced. Government reliance on general powers within the NHS Acts to conduct research became untenable as anti-fluoridationists argued that the initial study sites were nothing short of experimentation on human subjects without their consent, exactly as had been condemned in the recent Nuremberg Code. A subtle and rarely acknowledged shift to describing the study sites as ‘demonstrations’ not ‘experiments’, relying on NHS Act powers to prevent disease rather than to carry out research, only increased the suspicion of the burgeoning anti-fluoridation movement. It also did little to resolve key legal issues, since it remained the case that water providers had no statutory powers to add extra substances to water that was already fit to drink. Early opportunities to legislate were not taken amid an unsettled political climate, and the legal opportunity that this created was quickly seized on by those seeking to stop fluoridation in its tracks.

Legal action to prevent fluoridation only came to court on one occasion, and the judgment in *McColl* v. *Strathclyde* was quickly superseded by legislation, all of which gives the impression that it was only a small and rather unsuccessful aspect of the wider movement. As I have argued, however, this legal mobilization was in many respects a success, given that it provided a powerful disincentive for already beleaguered local authorities to press ahead with fluoridation. Though cases in Andover and Watford did not go to court and therefore offered no victory for either side, this, in fact, worked to the advantage of anti-fluoridationists by perpetuating uncertainty. Given that successive governments were only too aware that legal opinion was divided, if not pessimistic, as to whether fluoridation was lawful, this uncertainty could not be put to rest without new legislation. By raising publicly unanswered and unanswerable questions, these cases prevented advocates for fluoridation from promoting it more forcefully, and certainly discouraged many local authorities and water providers from pursuing fluoridation schemes of their own.

Anti-fluoridation campaigns are also a revealing example of politically diverse (and in some senses populist) legal mobilization, with campaigners coming from across the political spectrum. This shows that even in the 1950s, social movement legal mobilization was not confined to straightforwardly progressive causes. It was used to resist change by people anxious to protect individual rights to health and bodily autonomy, or collective rights to clean water and accountable public bodies, all of which seemed to be under threat. Questions and concerns about fluoridation were disseminated rapidly at the local level in particular, albeit with national organizations providing information and very possibly strategic input too. Conflicting views about what kinds of knowledge about fluoridation were reliable mapped onto conflicting views about who or what could be trusted and whose rights required protection. In the face of presentations of fluoride as sinister chemical waste and fluoridation as mass involuntary medication or simply unacceptable state interference in individual freedoms to decide what to consume or how to be healthy, competing claims about people’s rights to be protected from avoidable harms, or for communities to have access to fluoridated water even where some individuals disagreed, were slow to emerge. Legal mobilization was, in this context, an attempt to test and re-establish the trustworthiness of the state, using uncertainty over what constituted ‘wholesome’ water to raise larger concerns. By relying on their general powers under the NHS Acts to prevent disease and declining to propose new legislation, successive governments left fluoridation open to technical and moral challenges alike.

Though history rarely offers easily transferable lessons for the present day, this story provides a salutary reminder of the diverse causes to which rights claims can be put, and the effects of the law beyond courts and carefully calibrated decisions. In practice, the law and its uses – in government, as well as in social movements – are multi-layered, sometimes inconsistent, and propelled as much by circumstance as strategy. This echoes many cautionary tales about the value and uses of rights,^128^ and shows that legal mobilizations can be effective even without judgments or laws in their favour. One further lesson concerns the extent to which the initial framing of an issue can be difficult to dislodge. Anti-fluoridationists were quick off the mark in adopting the language of rights and spreading fears of medical experimentation, unreliable science, mass medication, and incompetent experts and bureaucrats. Those in favour of fluoridation were much slower to put forward the case for a shared responsibility to improve dental health, and the point that fluoridation would be of greatest benefit to those otherwise facing the worst dental health outcomes was rarely made. Had a more powerful case been set out from the beginning, backed by specific legislation to signal parliamentary endorsement, patterns of fluoridation (and tooth decay) across Britain may have been very different.

## Footnotes

1 K. Block, ‘Deep Structure and Controversy: Re-Reading the Fluoridation Debate’ (2009) 18 *Health Sociology Rev*. 246, at 246. See also G. Lowery et al., ‘When Evidence Alone Is Not Enough: The Problem, Policy and Politics of Water Fluoridation in England’ (2021) 17 *Evidence & Policy* 507; K Syrett, ‘Something in the Water: Opening the Public Health Law Policy Window for Fluoridation?’ (2024) 74 *Northern Ireland Legal Q*. 664; Nuffield Council on Bioethics, *Public Health: Ethical Issues* (2007) 119–139, at <https://cdn.nuffieldbioethics.org/wp-content/uploads/Public-health-ethical-issues.pdf>.

2 D. R. McNeil, *The Fight for Fluoridation* (1957); R. A. Freeze and J. H. Lehr, *The Fluoride Wars: How a Modest Public Health Measure Became America’s Longest-Running Political Melodrama* (2009); J. Johnson, ‘“A Man’s Mouth Is His Castle”: The Midcentury Fluoridation Controversy and the Visceral Public’ (2016) 102 *Q. J. of Speech* 1.

3 M. Brown et al., ‘Utah Bans Fluoride in Public Drinking Water, a First in the US’ *AP News*, 28 March 2025, at <https://apnews.com/article/utah-fluoride-ban-43f67153beb3e06ada9d782655fb15de>; M. Drachman and M. Diaz, ‘“I Am Very Proud of Utah”: RFK Jr Commends State Officials on Fluoridation Ban’ *ABC4*, 7 April 2025, at <https://www.abc4.com/news/politics/inside-utah-politics/utah-leads-nation-fluoridation-ban/>; American Dental Hygienists’ Association, ‘ADHA Supports Community Water Fluoridation amid Recent Challenges: American Dental Hygienists’ Association Warns Removal Would Harm Public Health, Increase Dental Disease Burden’ *American Dental Hygienists’ Association*, 9 April 2025, at <https://www.adha.org/newsroom/adha-supports-fluoridation-despite-challenges/>; American Dental Association, ‘Community Water Fluoridation Prevents Painful Dental Disease: Denying Public Health Measures to Americans Is Disgraceful’ *American Dental Association*, 28 March 2025, at <https://www.ada.org/about/press-releases/community-water-fluoridation-prevents-painful-dental-disease>.

4 S. Mian et al., *Water Fluoridation and Dental Health: 2024 Update* (2024) 4, at <https://researchbriefings.files.parliament.uk/documents/POST-PB-0063/POST-PB-0063.pdf>. This may be changing as a result of the Health and Social Care Act 2022: see Syrett, op. cit., n. 1.

5 Note, 9 March 1953, in The National Archives London (TNA) MH 58/531; letter from E. Russell-Smith to Mr Symon, Ministry of Housing & Local Government, 2 May 1953, in TNA MH 58/531.

6 A. Johnstone, ‘Battle Stations for New Fluoride War’ *Herald*, 19 August 1992, at <https://www.heraldscotland.com/news/12545486.battle-stations-for-new-fluoride-war/>.

7 M. Winstanley, ‘Fluoride: Scotland Bites Back’ *Times*, 13 July 1983.

8 *Herald*, ‘Obituary: Catherine McColl’ *Herald*, 26 May 2012, at <https://www.heraldscotland.com/opinion/13059339.catherine-mccoll/>.

9 For an excellent summary of popular knowledge about fluoride, see C. Sleigh, *Fluoridation of Drinking Water in the UK, c.1962–67: A Case Study in Scientific Misinformation before Social Media* (2021), at <https://royalsociety.org/-/media/policy/projects/online-information-environment/oie-water-fluoridation-misinformation.pdf>.

10 G. O’Hara, *The Politics of Water in Post-War Britain* (2017).

11 E. Lehoucq and W. K. Taylor, ‘Conceptualizing Legal Mobilization: How Should We Understand the Deployment of Legal Strategies?’ (2020) 45 *Law & Social Inquiry* 166, at 178.

12 S. R. Levitsky, ‘Law and Social Movements: Old Debates and New Directions’ in *The Handbook of Law and Society*, eds A. Sarat and P. Ewick (2015) 382, at 383.

13 C. Harlow and R. Rawlings, ‘“Striking Back” and “Clamping Down”: An Alternative Perspective on Judicial Review’ in *Public Law Adjudication in Common Law Systems: Process and Substance*, eds J. Bell et al. (2016) 301; M. W. McCann, ‘Legal Mobilization and Social Reform Movements: Notes on Theory and Its Application’ (1991) 11 *Studies in Law, Politics, and Society: A Research Annual* 225.

14 P. Blokker, ‘Using Liberal–Legal Tools for Illiberal Gains: The European Court of Human Rights and Legal Mobilisation by Conservative Right-Wing Actors’ (2024) 20 *International J. of Law in Context* 401.

15 C. Harlow and R. Rawlings, *Pressure through Law* (1992).

16 Id., p. 94.

17 S. A. Boutcher and H. J. McCammon, ‘Social Movements and Litigation’ in *The Wiley Blackwell Companion to Social Movements*, eds D. A. Snow et al. (2018) 306, at 311; C. Hilson, ‘New Social Movements: The Role of Legal Opportunity’ (2002) 9 *J. of European Public Policy* 238.

18 O’Hara, op. cit., n. 10, p. 215.

19 United Kingdom Mission, *The Fluoridation of Domestic Water Supplies in North America as a Means of Controlling Dental Caries: Report of the United Kingdom Mission, February–April 1952* (1953), in TNA MH 58/548.

20 Note on minute by G. Godber, 3 July 1952, in TNA MH 58/531.

21 Minutes, 18 June 1954, in TNA MH 58/533.

22 Records of events in Darlington are available in TNA MH 58/534.

23 A. C. Whipple, ‘“Into Every Home, into Every Body”: Organicism and Anti-Statism in the British Anti-Fluoridation Movement, 1952–1960’ (2010) 21 *Twentieth Century Brit. History* 330; O’Hara, op. cit., n. 10.

24 Sleigh, op. cit., n. 9, p. 17.

25 Id., p. 13.

26 Minute, 4 November 1952, in TNA MH 58/531.

27 Letter from E. Russell-Smith to Mr Symon, Ministry of Housing & Local Government, 2 May 1953, in TNA MH 58/531.

28 NHS (Scotland) Act 1947, s. 29. See also minute, 9 March 1953, in TNA MH 58/531.

29 Memorandum by E. Russell-Smith, 25 March 1955, in TNA MH 58/542.

30 Letter from Mr Mitchell to Mr Mottram, 25 September 1953, in TNA MH 58/531. See also minutes, 1955, in TNA MH 58/542.

31 Minute, 11 October 1956, in TNA MH 58/542.

32 Memorandum by E. Russell-Smith, 15 October 1956, in TNA MH 58/542.

33 Letter from R. E. Manningham Buller, AG, to Brian O’Brien, Ministry of Health, 7 February 1957, in TNA MH 58/542.

34 Compare, for example, memorandum, 14 March 1953, in TNA HLG 50/2858, and reference note number 9 by MoH to local authorities involved in the studies, July 1955, in TNA HLG 127/20.

35 Letter by H. J. L. Monk to the Town Clerk, Borough of Watford, 3 January 1956, in TNA HLG 127/20.

36 188 *HL Debs*, col. 456 (6 July 1954).

37 ‘Voters’ Veto’ petition, in TNA MH 58/534.

38 Letter by H. J. L. Monk to the Town Clerk, Borough of Watford, 3 January 1956, in TNA HLG 127/20; letters by Lord Douglas of Barloch to various correspondents, November 1959–April 1960, in TNA CAB 124/1641.

39 Memorandum by Dr W. G. Senior, 22 June 1951, in TNA MH 58/531.

40 Letter from MoH to Lord Douglas of Barloch, 26 November 1959, in TNA CAB 124/1641.

41 O’Hara, op. cit., n. 10, p. 230.

42 Minutes circulating around the MoH, June–November 1959, in TNA MH 58/549; minute, 18 April 1962, in TNA MH 58/549.

43 Opinions, 5 and 6 November 1962, in TNA MH 58/543.

44 669 *HC Debs*, col. 25 (10 December 1962).

45 668 *HC Debs*, col. 176 (26 November 1962); 245 *HC Debs*, col. 412 (10 December 1962); 681 *HC Debs*, col. 693 (17 July 1963).

46 ‘Conclusions of a Cabinet Meeting Held on 6 December 1962’, 6 December 1962, in TNA CAB 128/36/73.

47 O’Hara, op. cit., n. 10, p. 250.

48 MoH press release about the opening of the Hitchin Health Centre and Junior Training Centre, 17 May 1963, in TNA CAB 124/1641. Extracts from Powell’s speech can also be found in TNA MH 148/162.

49 Minute, 8 June 1961, in TNA MH 58/543, emphasis in original.

50 Block, op. cit., n. 1; Lowery et al., op. cit., n. 1.

51 Letters from A. Gill to Ministry of Health, 9 July and 5 August 1955, in TNA MH 59/534, emphasis in original. See also P. Clavell Blount, *Compulsory Mass Medication: A Factual Guide to the Fluoridation Issue* (1964) 9.

52 M. MacAulay et al., ‘Analysing the “Follow the Science” Rhetoric of Government Responses to COVID-19’ (2023) 51 *Policy & Politics* 466.

53 Confidential note, 10 October 1967, in TNA MH 148/153.

54 Memorandum by R. K. Alder, 19 July 1983 (reporting advice from 1978), in TNA JA 238/58.

55 Letter from SRC to Scottish Home and Health Department, 26 September 1984, in National Records of Scotland (NRS), HH58/181. See also M. Zander, ‘Jaws, or What’s in the Water’ *Guardian*, 1 July 1983.

56 D. Shaw, ‘Weeping and Wailing and Gnashing of Teeth: The Legal Fiction of Water Fluoridation’ (2012) 12 *Medical Law International* 11.

57 McCann, op. cit., n. 13, p. 230.

58 Whipple, op. cit., n. 23. See also minute, 13 November 1952, in TNA HLG 127/19; minute, 31 March 1955, in TNA MH 58/532.

59 J. Mew, ‘Chairman’s Report’ in *Housewives Today*, April 1956, in TNA MH 58/534. See also ‘Teeth versus Freedom’ in *Housewives Today*, April 1956, in TNA MH 58/534.

60 Whipple, op. cit., n. 23, pp. 342–346.

61 *Herald*, op. cit., n. 8.

62 G. A. Hosking, *Trust: A History* (2014).

63 Whipple, op. cit., n. 23, p. 346, p. 347. See also letter from ‘Eliza Doolittle’ to the editors, ‘When Does Water Become Medicine?’ *Northern Echo*, 11 February 1955, in TNA HLG 127/19.

64 Minute, 11 October 1956, in TNA MH 58/542.

65 Boutcher and McCammon, op. cit., n. 17, p. 310.

66 D. J. Borrett, *Something in the Water: The Anti-Fluoride Campaign in Andover 1955–1958* (2002) 47. See also newspaper clippings from the *Andover Advertiser*, 1955–1956, in TNA MH 58/537.

67 *Andover Advertiser*, ‘High Court Action against Borough Council?’ *Andover Advertiser*, 1 March 1957, in TNA MH 58/542.

68 Borrett, op. cit., n. 66, p. 66. See also minutes and memoranda, March 1957–July 1958, in TNA MH 58/542. For a useful summary of relator actions, see J. A. Jolowicz, ‘Protection of Diffuse, Fragmented and Collective Interests in Civil Litigation: English Law’ (1983) 42 *Cambridge Law J*. 222, at 239–242.

69 Borrett, op. cit., n. 66, p. 82.

70 Background note shared with Watford, undated but c. June 1963, in TNA MH 149/250.

71 Minute, July 1958, in TNA MH 58/542.

72 NPWA, *Annual Report 1961–2* (1962) 3, in Wellcome Collection (WC) PP/PCB/C/5/3.

73 Letter from Lord Douglas of Barloch to the British Waterworks Association, 19 June 1963, in TNA MH 149/250. As one civil servant drily noted, ‘[i]t would be unfortunate if this led to the disclosure that a Minister of Health had allowed fluoridation to continue even though he knew of the opinion at that time that it was illegal’: minute, 3 July 1963, in TNA MH 149/250.

74 Notes of meeting, 7 June 1963, in TNA MH 149/250. See also NPWA press release, 17 June 1963, in WC PP/PCB/C/5/3.

75 *Ryan* v. *Attorney General* [1965] IR 294; *Attorney-General; ex parte Lewis* v. *Lower Hutt City* [1965] NZLR 116. See also minutes and letters, February 1965, in TNA MH 149/251.

76 NPWA circular letters, 1 September and 23 February 1965, in WC PP/PCB/C/5/3.

77 *Watford Observer*, ‘Public Will Get No Say on Fluoride’ *Watford Observer*, 5 October 1990, at <https://www.watfordobserver.co.uk/news/nostalgia/15594471.Watford_suffers_a_crime_spree_in_October_1990/>.

78 Affinity Water now cover Watford and do not add fluoride to their supply, as of 5 June 2025.

79 Minute, 1 December 1964, in TNA MH 149/251. For complaints that local health authorities’ hands were tied, see letters from and to local health authorities, February 1965–February 1966, in TNA MH 148/167.

80 NPWA, *Chief Water Engineer on the History, the Wastefulness, the Negligible Dental Benefits and Doubtful Legality of Fluoridation. Also on the Impropriety of the Minister of Health’s Statement of 10*^*th*^ *Dec. 1962* (1963), in WC PP/PCB/C/5/3.

81 Letter from Dr V. P. McDonagh, Keighley Medical Officer of Health, to Dr E. Martin, MoH, 9 February 1966, in TNA MH 148/167.

82 British Waterworks Association circular under cover of letter from British Waterworks Association to Ministry of Housing & Local Government, 19 October 1962, in TNA HLG 50/2894.

83 Chief Medical Officer circular, 1965, in TNA MH 148/124.

84 Department of Health and Social Security, *Prevention and Health: Everybody’s Business – A Reassessment of Public and Personal Health* (1976) 63–64, 85. See also P. Clark, ‘“Problems of Today and Tomorrow”: Prevention and the National Health Service in the 1970s’ (2019) 33 *Social History of Medicine* 981; copy of the ‘Dobry opinion’, 12 April 1976, in NRS HH61/1383; NPWA newsletter, August 1976, in WC PP/PCB/C/5/1.

85 NPWA newsletter, April 1979, in WC PP/PCB/C/5/1.

86 Writ served 11 August 1978 (and copy of the defence), in NRS DD13/2620.

87 North Ayrshire Anti-Fluoridation Committee statement, 13 March 1969, in WC PP/PCB/C/1/43. See also newspaper clippings from the *Oban Times*, October–November 1970, in NRS DD13/2620.

88 Written question and answer ‘Fluoridation’, 974 *Hansard*, col. 43 (19 November 1979); minute, 21 September 1978, in NRS HH61/1383.

89 *Scotsman*, ‘Fluoridation for Some’ *Scotsman*, 18 March 1977, in NRS DD13/2620.

90 Minute, 19 October 1978, in NRS DD13/2620; *Glasgow Herald* ‘Summons Issued in Fluoride Battle’ *Glasgow Herald*, 2 November 1978, in NRS DD13/2620. See also correspondence between J. Wray and SRC, August–September 1978, in NRS HH61/1383.

91 Letter from P. Clavell Blount to E. Vaughan, 17 June 1988, in WC PP/PCB/C/1/43; correspondence between J. Stewart and P. Clavell Blount, undated but 1982–1983, in WC PP/PCB/C/1/43; Strathclyde Campaign against Fluoridation circular, 2 December 1980, in WC PP/PCB/C/1/43; NPWA, *Report of AGM*, undated but early 1980s, in WC PP/PCB/C/5/3.

92 Concern International letterhead, in WC PP/PCB/C/1/43.

93 R. Young’s report in Concern International circular, 2 October 1980, in WC PP/PCB/C/1/43.

94 B. Adhikari et al., ‘Trust Is the Common Denominator for COVID-19 Vaccine Acceptance: A Literature Review’ (2022) 12 *Vaccine* X 100213.

95 For an example of this counter-claim, see letter drafted by M. Senior, MoH, to British Medical Association, undated but c. August 1966, in TNA MH 148/168.

96 Shaw, op. cit., n. 56.

97 *McColl* v. *Strathclyde* [1983] ScotCS, Jauncey LJ, judgment.

98 Letter from J. McKay to K. Clarke, 21 September 1983, in TNA JA 238/58.

99 Committee paper under cover of letter, 21 September 1983, in TNA JA 238/58.

100 Harlow and Rawlings, op. cit., n. 13.

101 G. Stern, ‘Tails You Lose’ *Lincolnshire Daily Echo*, 6 July 1983, in WC PP/PCB/C/1/43.

102 McCann, op. cit., n. 13, p. 242.

103 Syrett, op. cit., n. 1, p. 674.

104 J. S. Sandman, *Response to Freedom of Information Request: Fluoride in Tap Water* (2010), at <https://www.whatdotheyknow.com/request/fluoride_in_tap_water#incoming-113151>; M. A. Lennon and S. C. Jones, ‘Fluoridation in Britain Today’ (1993) 306 *Brit. Medical J*. 666.

105 Borrett, op. cit., n. 66; O’Hara, op. cit., n. 10; Whipple, op. cit., n. 23.

106 84 *HC Debs*, col. 530 (24 October 1985). See also A. Page, ‘A Toxic System: Lord Douglas of Barloch and the Fight for “Natural” Food and Water (1940s–60s)’ (2018) 23 *Revue française de civilisation britannique*.

107 J. Hinton, ‘Militant Housewives: The British Housewives’ League and the Attlee Government’ (1994) 38 *History Workshop J*. 129; G. Love, ‘A “Mixture of Britannia and Boadicea”: Dorothy Crisp’s Conservatism and the Limits of Right-Wing Women’s Political Activism, 1927–48’ (2019) 30 *Twentieth Century Brit. History* 174.

108 Whipple, op. cit., n. 23.

109 *Kilmarnock Standard*, ‘Fluoridation in Kilmarnock, Ayrshire’ *Kilmarnock Standard*, 13 October 1962, in WC PP/PCB/C/5/3.

110 Letter from Mr Barron, Scottish Development Department, to Mr Renshaw, Department of Environment, 3 October 1978, in NRS HH61/1383.

111 *Herald*, op. cit., n. 8.

112 BBC News, ‘“Jack-the-Lad” MP’ *BBC News*, 3 March 2000, at <http://news.bbc.co.uk/1/hi/scotland/664995.stm>.

113 E. Gibbs, ‘Historical Tradition and Community Mobilisation: Narratives of Red Clydeside in Memories of the Anti-Poll Tax Movement in Scotland, 1988–1990’ (2016) 57 *Labor History* 439, at 441.

114 *Herald*, op. cit., n. 8.

115 Gibbs, op. cit., n. 113, p. 447.

116 McCann’s position is reported in a Strathclyde Campaign against Fluoridation circular, 2 December 1980, in WC PP/PCB/C/1/42. See also correspondence between P. McCann and the Scottish Home and Health Department, July–August 1979, in NRS DD13/3002; correspondence between P. McCann and the Scottish Home and Health Department, July–September 1979, in NRS HH61/1384; correspondence between McCann and the Speaker of the House and the Lord Advocate, December 1983–February 1984, in NRS HH58/181.

117 *Herald*, ‘Peter McCann’ *Herald*, 27 January 2004, at <https://www.heraldscotland.com/news/12518096.peter-mccann-former-lord-provost-of-glasgow-and-disabled-scot-of-the-year-who-campaigned-on-various-local-issues/>.

118 Sleigh, op. cit., n. 9.

119 Harlow and Rawlings, op. cit., n. 15.

120 This is evident in McCann, op. cit., n. 13. See also, more recently, L. Vanhala, ‘Legal Opportunity Structures and the Paradox of Legal Mobilization by the Environmental Movement in the UK’ (2012) 46 *Law & Society Rev*. 523; E. Kirk and K. Cruz, ‘Trade Union Legal Mobilization and Consciousness’ (2024) 51 *J. of Law and Society* S66.

121 See the Special Issue of *International J. of Law in Context* (2024) 20.3; the works mentioned in Boutcher and McCammon, op. cit., n. 17, p. 309.

122 T. Matthews, ‘Interrogating the Debates around Lawfare and Legal Mobilization: A Literature Review’ (2023) 15 *J. of Human Rights Practice* 24, at 25, 27, 40.

123 Blokker, op. cit., n. 14, p. 405.

124 See for example J. Stewart’s comments in her letters to P. Clavell Blount, undated, in WC PP/PCB/C/1/43.

125 Letter from Dr E. M. Senior to Dr Ludkin, 5 February 1965, in TNA MH 148/124; correspondence between Lord Barloch and Lord Hailsham, December 1959, in TNA CAB 124/1641.

126 K. Kocemba and M. Stambulski, ‘Populism, Non-State Actors and Right-Wing Legal Mobilization in Europe’ (2024) 20 *International J. of Law in Context* 297, at 297.

127 S. Kenny et al. ‘Introductory Overview’ in *Populism, Democracy and Community Development*, eds S. Kenny (2021) 1, at 5.

128 See for example C. Bob, *Rights as Weapons: Instruments of Conflict, Tools of Power* (2019).

